# Phenomenological study: the experiences of patients with nasopharyngeal cancer after undergoing chemoradiation

**DOI:** 10.1186/s12912-019-0356-9

**Published:** 2019-08-16

**Authors:** Ucip Sucipto, Agung Waluyo, Sri Yona

**Affiliations:** Faculty of Nursing Universitas Indonesia, Jalan Prof. Dr Bahder Djohan, Kampus UI, Depok, West Java 16424 Indonesia

**Keywords:** Chemoradiation, Nasopharyngeal cancer, Patient experience

## Abstract

**Background:**

Chemoradiation is a combination therapy of chemotherapy and radiotherapy. Because chemotherapy is given together with radiotherapy, the side effects are heavier and more severe for some patients. For nasopharyngeal cancer patients, the side effects involve nausea, vomiting, anorexia, diarrhea, mucositis, xerostomia, and tasting and hearing loss, which influence their quality of life. The purpose of this study was to explore the experiences of patients with head and neck cancer undergoing chemoradiation.

**Methods:**

A Phenomenogical desctipve, involving in depth interviewes was conducted during a 6 month study period. Eleven patiets with nasopharyngeal cancer participated in indepth inteviews Colaizzi’s method was used in analyzing data. The selection of participants in this study using purposive sampling method the inclusion criteria were1) the patient had been diagnosed with head and neck cancer, 2) minimum age of 18 years, and 3 had been received external chemoradiation, minimum 14 times of chmemoradiation. The study was conducted at one out-patientradiotherapy department. at Dharmais Cancer Centre Hospital, Jakarta.

**Result:**

The findings show three main themes: 1) xerostomia was the main physical complaint, 2) patients had decreased social interaction, and 3) having adequate support from the family was important for patients.

**Conclusion:**

The findings suggest involving family members when patients are undergoing chemoradiation. Adequate family support is needed to help the patients adapt to the side effects of chemoradiation with the best possible response.

## Background

Nasopharyngeal cancer is a malignant tumor that arises in the epithelium of the cavity behind the nose and is one of the most common types of carcinoma in the head and neck areas. The risk of head and neck cancer also increases in 30- to 50-year-olds and is more common in men than in women. There are several methods of treatment for head and neck cancer, such as chemotherapy, radiotherapy, surgery, and chemoradiation; however, surgery is rarely performed because of the nasopharyngeal anatomy, which is located behind the nose and is difficult to reach [1].

Choosing a treatment method for cancer is not easy. Many factors need to be addressed, such as the cancer itself, chemosensitivity or chemoresistance, the cancer cell population, the cancer growth cycle, the body’s immune system and therapeutic side effects [2]. One of treatment method is the combination of chemotherapy and radiotheraoy. It is the method of treatment performed in patients with an advanced stage of head and neck cancer and the presence of metastasis. The aim of chemoradiation is to control the tumor locally and increase the survival of head and neck cancer patients by way of systemic cancer treatment through microcirculation. Combined chemoradiation aims to control tumor cells, which has the benefit of inhibiting the regrowth of tumor cells that have been exposed to radiation [3].

The method of healing depends on the type of head and neck cancer, its level of ferocity, and the stage of the cancer itself. In advanced cancer, where the cancer has metastasized or spread, healing is usually accomplished through the combination of several methods of treatment, such as chemoradiation, while healing by using a method such as radiation alone is done when the tumor is localized and small. The selection of chemoradiation as the primary choice of treatment for nasopharyngeal cancer is primarily based on the fact that most (75–95%) head and neck cancers of undifferentiated carcinomas (WHO type 3) and non-keratinized carcinomas (WHO type 2) are highly radiosensitive [4]. Another reason is the anatomical factor of nasopharyngeal cancer, which is located at the base of the skull with many vital organs, requiring extensive surgery to obtain a free margin [5].

Radiotherapy is a medical action that uses ionizing radiation to kill as many cancer cells as possible with as little damage to normal cells as possible. Radiotherapy has side effects that must be theoretically experienced by all sufferers. These side effects occur as a result of radiation exposure to the epithelium, support tissues, and vascular systems. Examples of these side effects include mucositis, xerostomia (hyposalivation), changes in tasting ability, dysphagia, and dysphonia.

Side effects of radiotherapy that are slow to appear require special attention because the damage is generally permanent. This effect occurs in cells that divide slowly. Most slow side effects on the head and neck areas occur within three years after treatment. These slow side effects include tooth decay; soft tissue necrosis; bone necrosis; cartilage necrosis; damage to the eyes, ears, and nerves; ischemia, and fibrosis. The incidence of acute side effects results in organ function disorders. Post-radiotherapy patients suffer from dry mouth, which directly intereferes with chewing, swallowing, and talking. All of these radiotherapy side effects will have an impact on the quality of life and physical status of head and neck cancer patients [6].

Based on World Health Organization’s data, nasopharyngeal cancer ranks 24th as the most common malignant incidence in the world. In 2002, the International Agency for Research on Cancer (IARC) found around 80,000 new cases of nasopharyngeal cancer worldwide, where 40% of the death cases are from China.

In Indonesia, Nasopharyngeal cancer ranks 4th from all malignant diseases after cervical cancer, breast cancer, skin cancer and is the most common cancer in the head of the neck. Nasopharyngeal cancer is the first rank of incidence in the field of Ear Nose Throat Surgery Head and Neck [7]. Based Medical records of Dharmais Cancer Hospital, it isestimated 903 new cases of patients with Nasopharyengal during the period 2012–2016, with an average of 181 people.

There are several treatment for KNF patients, such as chemotherapy, radiation, surgery and chemoradiation.. Chemoradiation theraphy is a given for patient with KNF, together with radiation theraphy. The aim of chemoradiation is to control the tumor locally and increase the survival rate of nasopharyngeal cancer patients [8]. Radiation is considered as the primary treatment for nasopharyngeal cancer. The reason for this is that nasopharyngeal anatomy factor that lies at the base of the skull with many vital organs. Consequently, obtaining tumor-free margin is difficult when extensive surgery is performed [9].

Many previous study found that several side effect of radioteraphy may occur for patients, such as, such as mucoositis, skin reaction, loss of taste sensation, xerostomia and dysphagia [10]. This side effect should be treatmented as soon as possible to prevent further malfunction patients that can lead to poor quality of life patient. Thus, to provide better treatment, it is essential to undertand how the life experience of KNF patient undego chemoteraphy. However, few stud has been conducted about the life experience KNF pasien in Indonesia.

Therefore, theresearch question in this paper is “How was the experience of nasopharigealn patients after undergoing chemo-radiation”.

## Method

This study used a phenomenological approach, to explore the life experiences of being nasopahringeal patients undergoing chemoradiation. The following questions facilitated the exploration: what do feel about having chemoradiation? Do you experience any side effects of theraphy? What factors facilitatate or help you in dealing with side effect of chemoradiation?. This research has been approved by the ethics committee of the Faculty of Nursing, University of Indonesia, with proposal no. 146./Un2.F12.D/HKP.02.04/2017.

### Participants and procedure

Participants were recruited from one out patient unit at Dhamais Cancer Center, Jakarta. purposive sampling method Was used, the inclusion criteria: 1) the patient had been diagnosed with head and neck cancer, 2) the patient had a minimum age of 18 years, and 3) the patient had received external chemoradiation at least 14 times. Each participants engaged in a face to face, tape recorded, in depth interview. The interviews were conducted in a private, quiet small room in the hospital. The interviews lasted 40 min to 70 min. There was no follow up interview. All coding was read several times by the first and and the third author. For final coding, team research met and discuss each coding, and to determine final themes, thus enhancing trustworthiness in this study.

## Result

### Characteristics of participants

The study involved 11 participants, predominantly male (6, or 55%). Almost all were above 35 years old (10, or 91%). Many were in high school (5, or 45%) and had been diagnosed with a cancer in the last year (45%), while some were diagnosed within the last two years (18%). The majority’s religion was Islam (82%), and more than 50 % did not have a regular job (64%) and worked as housewives, retired civil servants, and college student. Details of the participants’ characteristics can be found in Table 1.

**Table 1:** of the Characteristics of Participants

Participants	Gender	Age	Education	Years of diagnosis nasopharyngeal Cancer	Religion	Job
P1	Female	37	Junior High School	2016	Moeslem	Traderers
P2	Female	61	DIPLOMA	2016	Christian	Retired
P3	Female	18	Senior High School	2016	Moeslem	Student
P4	Female	39	Junior High School	2013	Moeslem	Traderers
P5	Male	37	Senior High School	2017	Moeslem	Laborers
P6	Male	52	Junior High School	2014	Moeslem	Neighborhood chief
P7	Male	61	Diploma	2016	Christian	Retired
P8	Female	48	Magister	2017	Moeslem	Lecturer
P9	Male	44	Senior High School	2015	Moeslem	Neighborhood chief
P10	Male	35	Senior High School	2015	Moeslem	Laborers
P11	Male	49	Senior High School	2016	Moeslem	Laborers

According to the results of interview analysis, researchers have identified several themes related to the purpose of research. The themes are: 1) Xerostomia is the main physical complaint of participants, 2) Decreased social interaction, 3) The existence of adequate system support from the family.

### Theme 1: xerostomia was the main complaint of the participants

Xerostomia was mainly reported by participants as the physical experience after chemoradiation.

The first theme is formed from two categories, the decrease of saliva production and the increase of mineral water consumption. Xerostomia experienced by participants is characterized by decreased production of saliva, dry mouth, difficulty swallowing food, and constant thirst. Other physical complaints perceived by the participants are impaired skin integrity and sensory changes. In order to support this theme, here below are some quotes which are taken from interviews:

The findings showed that there were two categories for this theme: decreased saliva production and increasing water consumption as one way to keep rehydrate.

Increasing water consumption as one way to keep rehydrate.

The majority of participants had complaining about Increasing water consumption as one way to keep rehydrate. In order to support this theme, here below are some quotes which are taken from interviews:

“*I am feel thirsty” (P1, line 6)*

“*I am want to drink continuously” (P2, line 32)*

*“I was eat liquid food” (P7 line 38)*

### Decrease saliva production

The majority of participants had complaining about feeling thirsty and dry mouth due to decrease salova production. For example:

*“My mouth and throat mucous membranes feel so dry.” (P2)*

*“I am a thirsty man.” (P1, line 8)*

*“I feel that I have less saliva production. It makes me so dry.”(P3, line 7)*

*“just like it’s so thick, so the saliva looks like it’s thick, from the 15th ray to the present (P1, line 35)”*

*“I feel that myThroat is dry, want to drink, day and night. (P2, line 8)”*

*“Can chew but can not swallow it because sticky there is no saliva.” (P5, line 24)*

### Theme 2: patients had decreased social interaction. This theme illustrated that participants had a lack of confidence due to physical changes as an adverse effect of chemoradiation

Decrease in social interaction is a disturbance of interaction that occurs either between individuals or between groups This theme the feeling of not confidence category. Participants reported that they experienced the decreased social interaction due to chemoradiation therapy. In order to support this theme, here below are some quotes which are taken from interviews:

*“Usually just alone, self-seal, self-cage, need time to be alone, withdraw themselves in their own room and just be silent” (P3, line 47)*

*“I am Just want to be alone. Do not want to hang out with other. My physic has changed now. Feel different now.” (P7, line 8)*

*“Usually, confine themselves in the room, need time alone, just confine themselves in the room, silent (P3, line 47)”*

*“when I was healthy, I spend more time outside the house. Then, Once I am sick, I barely go outside anymore. I spend more at home (P6, line 102)”*

*“I rarely going out because people seems pay attention to my physic and say that I look slim and pale (P6, line 107)”*

### Theme 3: having adequate support from the family was important for patients

The existence of adequate system support from the family makes the participants become stronger and more consistently motivated to undergo chemoradiation. In data analysis, this theme is determined by category of the family support. This can be seen from several statements of participants below:

*“My husband supports me. If no one supported me, I would feel stressed. I can feel calm; you know why? Because step daughter always supports me too by asking me about how my treatment [is] and how my sleep [was].” (P2, line 90)*

*“If I am going out to talk with other, my wife always accompanies me, because I have hearing problem. If I can’t hear what people say, my wife will help me by whisperin to my ear (P6, line 102)”*

*“my husband takes care of me. He always accompanies me if I am going to see doctor or I will go to get some treatment (radiation) (P4, line 94)”*

*“My family, who lives in Java (difference city), always call me and ask about my health condition (P3, line 140)”*

## Discussion

### Xerostomia is the Main complaint of the participants

Of the adverse effects of radiation, xerostomia was the the main problem that affected the health of nasoharynegal cancer patients post-therapy.

In this study, participants experienced severe side effects caused by chemoradiation treatment. The most common side effect of chemoradiation in this study was xerostomia. All participants who experienced xerostomia were characterized by a decrease in salivary production, dry mouth, difficulty swallowing food and even fluids, and constant thirst. Xerostomia is the decrease in salivary production caused by the destruction of salivary gland structures due to radiation exposure with varying degrees of damage to the exposed salivary glands. The amount and severity of salivary glandular tissue damage depends on the dose and duration of irradiation. The saliva produced by the salivary glands in the early stages becomes more viscous with acute inflammation, and then the salivary glands become atrophied and fibrotic. During radiation, serous acinar cells are affected first before mucous acinar cells. As a result, saliva becomes more sticky and thick. Saliva production decreased by 50% one week after radiation. Changes in salivary composition also occur, including decreased immunoglobulin A secretion and buffer capacity, salivary pH becomes acidic, and others [11].

The research conducted by Thsalis [11] indicated that there is a significant decrease in salivary production of patients with radiation to the head and neck areas. This is because when those areas are irradiated, the salivary glands are exposed to radiation in the same dose and volume as the primary tumors, which can damage the cells in the salivary glands so that saliva production decreases. Salivary production will decrease significantly if the dose of radiotherapy increases. This is due to the deterioration of acinar cells in the salivary glands, especially the parotid glands. These cells are very sensitive to radiation. Involving salivary glands in the radiation area can cause fibrosis, fat degeneration, atrophy of acinar cells, and necrosis of glandular cells [12].

Another major physical complaint experienced by participants in the study was changes in tasting. Changes in participants’ ability to taste are a direct effect of irradiation on tasting receptor cells. In this study, some participants (P2, P3, P4, P6, P9, and P10) experienced sensory changes, marked by an inability to taste food as sweet, salty, sour, or other tastes. They could only taste food as bitter. This happened because tasting receptors are radiosensitive. When they are exposed to continuous radiation, the taste receptors underwent structural changes. Increased flow viscosity and salivary biochemical structures make saliva act as mechanical barrier, which makes it more difficult for the tongue to come into contact with food surfaces.

In his research, Gautama et al. mentioned that there are differences in the sense of taste and smell of nasopharyngeal cancer patients compared to other head and neck carcinoma patients who receive radiation therapy. Changes in taste and smell function often occur after radiation therapy and chemoradiation in patients with head and neck carcinomas, including nasopharyngeal carcinoma. Changes in chemosensors are induced by local and systemic effects of radiation, such as cell damage, receptor disorders, nerve changes, or taste receptor changes. Radiation can disrupt chemosensory receptors through mucous induction and causes direct damage to receptors as well as nerve connections [13]. In this study, only a small percentage of participants experienced an interruption in their sense of smell, such as nasal congestion and secretion that disturbed their daily activities, as revealed by P6, P9, and P10.

### Limited social interaction

In this study, the participants felt a decrease in self-confidence after undergoing chemoradiation. Feelings of insecurity were experienced by the participants. They confined themselves in their house, spent more time at home, did not get along with others, did not go out, and withdrew themselves. In this study, most participants did not feel confident because of physical changes that occured after undergoing chemoradiation, such as blackening skin around the neck, continuous secretions from the nose, lumps in the neck, and hearing loss. Research conducted by Wahyuni, Huda, and Utami [14] states that cancer patients who undergo cancer treatment such as chemotherapy will experience a decrease in self-confidence due to the physical changes that occur in patients. Nasopharyngeal cancer and the exhausting treatment process produce a state of stress and emotional burden, that cancer sufferers feel insecure and worried about communication problems with their family [15]. In line with that opinion, Damayanti, Fitriyah, and lndriani revealed that in general, head and neck cancer patients have problems with social interaction, withdrawal, and communication. In this study, most of the participants came from outside the city of Jakarta and some even from outside the island of Java, thus forcing participants to split up and leave their families due to the treatment process [16]. There were some participants who had to rent a house or board behind the Dharmais Cancer Center Hospital in order to save time and effort, as was done by P1, P2, P3, and P7. Because radiation should be done every day, five days per week (except Saturday, Sunday, and holidays), participants chose to stay around Dharmais Cancer Center Hospital. This kept participants away from their families, which can lead to a lack of a family support system and can increase the risk of death. This matter emphasizes that social relationships improve physical health status [17].

### Adequate support system from family

Several studies have identified family support as an essential part of nasopharyngeal cancer patients coping with their condition. Family support can become one of the main coping resources for positive adjustment to the adverse effects of chemoradiation. Due to the effects of this treatment, participants needed social support from the people arround them, particularly from their families.

According to Muhamad, Afshari, and Kazilan, head and neck cancer patients receive the most support from spouses, children, parents, and relatives, who assist them primarily in making decisions, providing emotional support, motivating and inspiring, providing information, providing spiritual guidance, and providing facilities [18]. In line with that opinion, Gorman revealed that a favorable system of support for cancer patients can give them a more positive mind-set and can help in predicting the extent of participants’ healing. In this study, all participants mentioned that they received family support, and it was helpful for them in adjusting to their physical and psychological changes during and after chemoradiation. Their families’ support gave them strength and a positive spirit when undergoing chemoradiation treatment [19].

In accordance with the results of the research conducted by Saragih on head and neck cancer patients undergoing chemoradiation and who received emotional support from their families, 52% patient received emotional support from their families. The presence of support from the family makes the patient feel not alone and reduces the sense of burden because they can share their feelings with their families. Family presence can help participants with their emotional mastery during chemoradiation treatment. In this study, all participants received adequate support from family, such as always being accompanied by family during radiation; good communication with family living outside the city; and support from friends, relatives, and the environment [20].

Clark (2005) also revealed that support systems are a decisive factor in successfully overcoming insecurity. Support systems become a psychological factor for head and neck cancer patients to help them forget the negative aspects of their treatment and think more positively about their environment. According to Bomar, support systems are a form of serving behavior from families and relatives, whether through emotional support (e.g., attention, compassion, empathy), award support (e.g., appreciation, feedback), information support (e.g., sugestion, advice, information), as well as instrumental support (e.g., aid of funds, manpower, and time). Participants who receive adequate support feel comfortable, cared for, and not alone in undergoing chemoradiation [21].

## Conclusion

The findings of this study suggest that nurses need to pay attention to xerotemia as the main physical effect of nasopharyngeal cancer patients post-therapy. In addition, receiving adequate family support was found to be a positive coping mechanism during chemoradtion therapy. Therefore, it recommended that nurses should provide appropriate education to minimize ongoing adverse effects and support the use of effective coping mechanisms, including involving family member during chemoradiation therapy.

## Data Availability

Due to privacy data, all data and material is available if requested.

## Abbreviation

WHO: World Health Organitation
